# The paradox of acceptance: A content analysis of Iranian married female adolescent in confronting pregnancy

**DOI:** 10.1371/journal.pone.0216649

**Published:** 2019-05-10

**Authors:** Maryam Moridi, Farkhondeh Amin Shokravi, Fazlollah Ahmadi

**Affiliations:** 1 Department of Health Education and Health Promotion, Faculty of Medical Sciences, Tarbiat Modares University, Tehran, Iran; 2 Department of Nursing, Faculty of Medical Sciences, Tarbiat Modares University, Tehran, Iran; Kwame Nkrumah University of Science and Technology, GHANA

## Abstract

Adolescent pregnancy is a major health problem significantly associated with adverse effects on the health of both adolescents and their newborns. However, there is a lack of clarity over adolescent pregnancy from adolescents' perspective, particularly in the low- and middle income countries including Iran. This study aimed to explore the experiences of Iranian married female adolescent in confronting pregnancy. This qualitative research was conducted using conventional content analysis approach. Unstructured interviews with 24 female adolescents (aged 14–18 years) were used for data collection, and data analysis was done simultaneously. In this study, the participants were recruited from urban and rural primary healthcare centers (PHCs). The PHCs were selected randomly from several towns and villages of Guilan Province, Iran. The participants were recruited through a purposive sampling method. After data analysis, four categories were extracted from 24 interviews, including "actively accepting pregnancy", "passively accepting pregnancy", "hope and satisfaction" and "frustration and regret". These categories formed the main theme of "paradox of acceptance" in confronting pregnancy. The concepts that have emerged based on the results of the study can potentially help develop comprehensive and age-tailored health promotion programs to confront pregnancy more successfully for female pregnant adolescents. Further research, particularly on the long-term effects of teenage motherhood is suggested.

## Introduction

Adolescent pregnancy is reported as a public health concern in many countries [1]. Annually, about 16 million female adolescents (aged 15–19 years) give birth globally [2]. Added to this, approximately one million births occur to females younger than 15 years; 95% of these births take place in the low- and middle-income countries [1]. The minimum legal age for males and females to marry in Iran is 15 and 13 years old, respectively. However, if the three conditions including the father's permission, best interests of the child and confirmation of competent court are met, the age of marriage will be lower. Iran's population of adolescents is high, and the age specific fertility rate in this group has been raised from 25 to 35 per 1000 adolescents from 2010 to 2011 [3]. Iran's population policy has shifted from a decrease to an increase in the consequent decades [4]. Therefore, it is predicted that the fertility rate of Iranian adolescents will be elevated by 2025. It is noteworthy to mention that, due to the cultural context based on Islam in Iran, females give birth during the first year of marriage [5], so they are confronted with simultaneous events such as marriage, pregnancy, and mothering in the transition to adulthood.

Adolescent mothers and their children have higher rates of health problems and negative outcomes, including medical complications, lower educational achievement, and socio-economic consequences. In addition, adolescent pregnancy is perceived as an undesirable social phenomenon [6, 7]. However, a number of quantitative studies on unmarried pregnant adolescents have revealed that adolescents have positive attitudes toward early childbearing [8–10] such as generating a connection to their parents and family, happiness, fulfilling gender roles [11], accountability, maturity, autonomy, and a meaningful life [12]. Due to conflicting perceptions about adolescent pregnancy, it is essential to investigate the consequences of pregnancy from the perspective of adolescents with a special focus on the role of socio-cultural factors influencing adolescents' feelings and choices. Understanding the adolescents' perspectives on pregnancy, through qualitative approaches, can help gain better insight into the challenges of adolescents in confronting pregnancy. However, while most pregnancies in Iran occur within the marital relationship, a majority of qualitative studies are done with unmarried female pregnant adolescents and indicate that these adolescents have mixed feelings about their pregnancy. Furthermore, a phenomenological study from a southern city of Iran on married female adolescents reported that pregnancy in adolescents resulted in the fast development of physical, psychological, social, and spiritual aspects of these individuals [4]. Consequently, by considering the complexity and diversity of the phenomenon of adolescent pregnancy in the context of time and place, the researchers aimed to explore how married female adolescents living in Guilan, Iran confront and accept their pregnancy.

## Methods

### 2.1 Study design

Due to the nature of the study, the qualitative content analysis method was used. Content analysis is a systematic approach that provides new insight into a particular phenomenon. It leads to valid deduction from data and is appropriate for exploring the experiences and views of people towards the issue of interest [13]. The current study was conducted from November 2015 to October 2016 in Guilan Province (in the north of Iran).

### 2.2 Study settings

In this study, the participants were recruited from urban and rural primary healthcare centers (PHCs). All PHCs and all available eligible females were selected from several towns and villages of the Guilan Province using census method.

### 2.3 Study population

The inclusion criteria were married females aged 14–18 years, being pregnant, living in the Guilan Province, registered to the PHCs or study hospital, speaking and understanding the Persian language, and willing to participate in the study.

### 2.4 Sampling approach

At first, 24 female adolescents who met the inclusion criteria and had been registered in the antenatal clinics of PHCs or the referral obstetric hospital in Rasht City (the capital of Guilan Province) were recruited using a purposeful sampling method. For this purpose, the telephone numbers of potential participants were selected from registry lists. Next, potential participants were contacted and provided with information about study. As soon as the female adolescents agreed to participate, the place, time and date of the interview were determined at their convenience.

### 2.5 Data collection

In total, from all the PHC's, 30 female pregnant adolescents were eligible to participate in the study. Of these 30 females, three were reluctant to participate, two did not have their family's consent, and one could not speak Farsi. Individuals who were interested in participation were then provided with details about the objectives of the study. The informed consent was obtained from the adolescents and legally acceptable representative or adult separately. The data for the present research was collected using unstructured, in-depth and one on one face-to-face interviews with 24 participants conducted by the first researcher who was supervised by two other experts. Duration of the interview sessions varied from 30 to 90 minutes. The interviews were conducted in PHCs and/or in more convenient locations such as the participants' homes at their request.

At the beginning of the interview, the participants were asked a general question: "Would you please explain your experiences of pregnancy?" or "Please tell me how you felt when you found out you are pregnant?". These questions were then followed by probing questions (e.g. "What do you mean exactly?" or "Would you please explain more?") for more clarity and insight into each response.

### 2.6 Data analysis

All interviews were audio-recorded in mp3 format and transcribed verbatim in Persian immediately after each interview prior to the next interview. Each transcript was imported to MAXQDA 10 software for data analysis. Concurrent with data collection, data analysis was done using Graneheim and Lundman's method (2004) including, reading the entire transcription of the interview to achieve an overall understanding of its content, specifying semantic units and basic codes, classifying initial similar codes in more comprehensive categories, and ultimately, extracting more abstract themes from the categories [14]. Data saturation was obtained after interviewing 21 participants when no new concepts or categories were developed thereafter. We elevated the sample size to assure the data saturation.

### 2.7 Translation

All processes of conducting interviews, coding, extracting primary codes, sub-categories, categories and theme, writing interpretations and writing the manuscript were done in Persian language by the authors. The manuscript was translated to English in four steps. First the authors wrote the primary first draft of the manuscript, then the draft with the Persian version was sent to an academic English institute for professional translation, next it was sent to an academic English editor. Finally it was reviewed and revised by an American native English editor living in Iran. After that, it was again sent to a native English editor living in the USA.

### 2.8 Trustworthiness

Four criteria (credibility, confirmability, transferability and reflexivity) are used to evaluate trustworthiness or rigor in a qualitative study [15]. The following strategies were used to ensure trustworthiness in this study. First, according to the study question, the conventional content analysis method was employed under careful supervision of the research team. Second, all interviews were conducted with a trained interviewer in conducting qualitative research and interviewing techniques. Third, the interviewer had a prolonged engagement with the data. The researcher developed trust and connected with the participants, considering a variety of perspectives, and co-constructing the meaning of adolescence. The first eight interviews were coded independently by each author, after which the interpretations were compared. If the interpretations differed, they were discussed until consensus was obtained. At the end of the study, member checking and peer check methods were used. In the member check method, the printed transcribed file was returned to participants to match the accuracy of the data with their experiences. In the peer check method, the transcripts, codes and categories were sent to four impartial professors in nursing, public health and social sciences to review the credibility of the extracted categories and sub-categories. Fourth, the researcher tried to choose participants with maximum variation in females' age, from extended and nuclear families and urban and rural settings. Finally, in addition to audio-records and transcripts, multiple data sources, including field notes, observation, memo, and diaries were used.

### 2.9 Ethics

This study was a part of a doctoral dissertation, which was approved by the Ethics Committee of the Research Deputy at Tarbiat Modares University. In addition to this approval, each participant and her spouse or her family member signed a written informed consent letter before the interview.

## Results

Most of the participants were in the age range of 17–18 years (n = 13); three of them were 14 years old and eight were 15–16 years old. All of the participants were housewives with primary school to high school education and all of them were Muslim; most of them were pregnant for the first time during the interview (Table 1).

**Table 1:** Characteristics of the participants.

Participant's code	Age	Gestational age	Gravidity	Parity	Duration of marriage	Educational level	job	Husband's age	Husband's educational level	Husband's job
1	17 yr	41 wk	1	0	2 yr	8 yr	HW	32 yr	11yr	SE
2	16 yr	28 wk, 4d	1	0	4 mn	11 yr	HW	31 yr	11yr	SE
3	15 yr	39 wk	1	0	2yr	10 yr	HW	24 yr	11yr	SE
4	16 yr	40wk+2d	1	0	1 yr	6 yr	HW	21 yr	11 yr	SE
5	17 yr	38 wk	1	0	2 yr	9 yr	HW	24 yr	7 yr	SE
6	14 yr	36 wk	1	0	1 yr	6 yr	HW	24 yr	11 yr	SE
7	17 yr	37 wk	1	0	10 mn	5 yr	HW	20 yr	14 yr	US
8	17 yr	39 wk, 2d	1	0	1 yr	6 yr	HW	26 yr	9 yr	SE
9	17 yr	38 wk. 1d	2	0	4 yr	5 yr	HW	24 yr	11 yr	SE
10	15 yr	27 wk, 2d	1	0	10 mn	8 yr	HW	22 yr	11 yr	SE
11	16 yr	16w, 5d	1	0	2 yr	8 yr	HW	25 yr	10 yr	SE
12	18 yr	14 wk	1	0	7 mn	8 yr	HW	19 yr	11 yr	SE
13	18 yr	18 wk	1	0	2 yr	8 yr	HW	21 yr	5 yr	SE
14	17 yr	29 wk	2	1	4 yr	10 yr	HW	23 yr	11 yr	SE
15	16 yr	36, 4d	1	0	1 yr	5 yr	HW	19 yr	6 yr	SE
16	16 yr	32 wk, 3 d	2	1	3 yr	7 yr	HW	22 yr	8 yr	SE
17	14 yr	22 wk	1	0	1 yr	9 yr	HW	24 yr	16 yr	SE
18	17 yr	13 wk, 2d	1	0	1 yr	9 yr	HW	22 yr	8 yr	SE
19	17 yr	19 wk, 4d	1	0	1 yr	7 yr	HW	20 yr	8 yr	SE
20	16 yr	35wk, 6d	1	0	2 yr	8 yr	HW	27 yr	16 yr	SE
21	18 yr	31wk, 2d	1	0	1 yr	7 yr	HW	25 yr	13 yr	US
22	18 yr	26 wk, 3d	2	0	3 yr	6 yr	HW	22 yr	9 yr	SE
23	17 yr	8 wk	1	0	6 mn	10 yr	HW	24 yr	7 yr	SE
24	14 yr	21 wk	1	0	1 yr	6 yr	HW	28 yr	11 yr	SE

**Fig 1:**
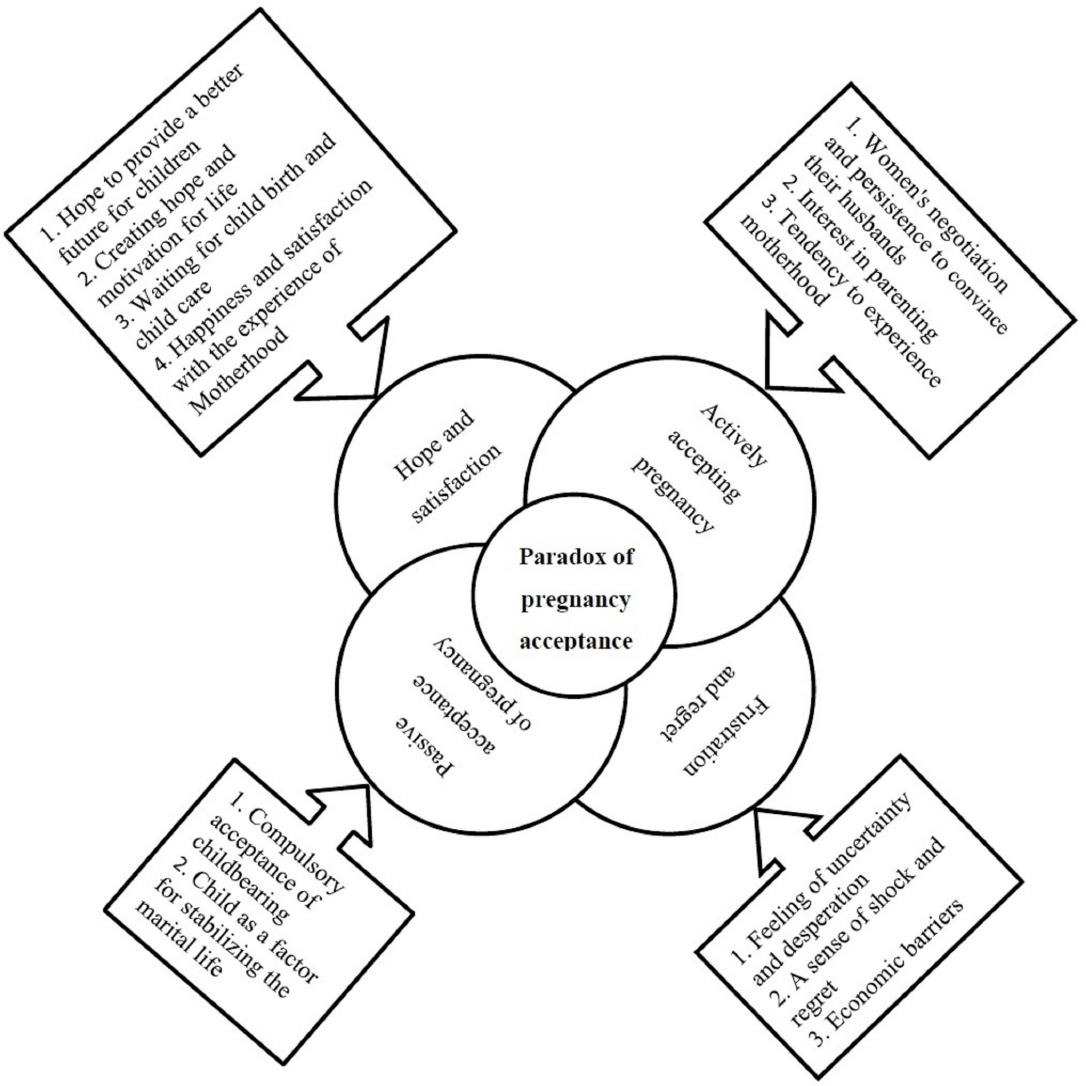
The categories and sub-categories of the paradox of pregnancy acceptance.

In total, 980 primary codes were extracted from 24 interviews, and were then compared to one another and categorized in 12 sub-categories. Next, the 4 categories, which included the active acceptance of pregnancy, the passive acceptance of pregnancy, feelings of hope and satisfaction, and feelings of frustration and regret, emerged from them and formed a main theme of "paradox of pregnancy acceptance". The categories and sub-categories are depicted in Fig 1.

The paradox of accepting pregnancy is the early response of young female to pregnancy. Usually, the decision for planned childbearing was established before pregnancy and for unplanned ones after the pregnancy in active or passive forms. In the active form, it is regarded as deciding to accept and undertake the responsibilities of pregnancy egocentrically, and in the passive form, it is characterized by the family's and husband's compulsion and insistence without regard to the females' desire for a child. Based on the kind of acceptance (active or passive), female adolescents are exposed to the dual feelings of hope and satisfaction, and frustration and regret when it comes to their experience of pregnancy. Generally speaking while these conflicting feelings exist simultaneously in both groups, hope and satisfaction were predominant in the teens who accepted pregnancy actively and frustration and feelings of regret were predominant in those who accepted it more passively. The degree of dominance of each of them was determined by either an egocentric (thinking only about herself and what is good for her) or an allocentric (having one's interest and attention centered on other people) decision about childbearing.

### 3.1 Actively accepting pregnancy

The majority of the participants expressed that they decided to become pregnant completely egocentrically. The reason for this decision was a desire to experience motherhood and an interest in parenting which led the teen to persuade her husband to accept her decision about becoming pregnant. This category includes three sub-categories: Females' negotiation and persistence to convince their husbands, Interest in parenting, and Tendency to experience motherhood.

#### 3.1.1 Females' negotiation and persistence to convince their husbands

Some of the participants mentioned using indirect negotiations as they tried to prepare their husbands to accept the pregnancy and its responsibilities. In other words, if the females were inclined to have a child but theirs husbands seemed reluctant, they tried to convince them otherwise using different approaches. Ultimately, these negotiations involved the cooperation and agreement of the couple regarding this decision:

*“I really wanted to have a child*. *However*, *once I saw that my husband was not interested*, *I tried to speak with him about the issue*. *I said that we have been married for a few years and we currently have a house and a car and can take care of the expenses related to the child*. *With this*, *I was able to convince him and he agreed to have a child*.*”* (Participant 13)

*"At first*, *my husband did not accept my pregnancy*, *so he pushed me to abort the baby* …*; so I tried to satisfy him and told him that all of your friends have two babies*, *I want just one*! *I repeated my words several times and ultimately convinced him*.*"* (Participant 23)

#### 3.1.2 Interest in parenting

Some participants mentioned that they accepted the pregnancy because of personal interest in childbearing and parenting. They also expressed that this interest existed even before their marriage and they were inclined to experience parenting again as a result of taking care of their younger siblings. These adolescents recognized the desire and interest in having a child as the axiom of life and believed that every human would subconsciously love and protect his/her child. In this respect, some participants said:

*"As both my husband and I would love to have a child*, *I just wanted to get pregnant* …*; I have really loved children since my childhood*.*"* (Participant 4)

*"I love babies*, *I took care of my brother myself*. *A child is sweet*.*"* (Participant 17)

*"When my sister was born I cared for her myself*. *When I look at a child*, *I have a good feeling; kids are cute*, *like my sister*.*"*(Participant 6)

#### 3.1.3 Tendency to experience motherhood

Based on the data collected, the desire to experience motherhood was one that was shared among all the participants:

"*I felt so happy about the fact that I was pregnant and that I would give birth to a child and become a mother soon*. *When I hear other mothers talking about their children*, *I always want to be able to experience the same feelings*. *For example*, *when they talk about buying their children clothes*, *or the fact that they miss them when they aren't around*." (Participant 18)

*"I like my pregnancy so much*. *When my friend was pregnant and her baby was born and she hugged him*, *I was jealous of her child*.*"* (Participant 3)

### 3.2 Passive acceptance of pregnancy

Some participants mentioned that, while being pregnant was against their personal desires, they accepted it due to the pressure they felt from their families. This category included the two subcategories of compulsory acceptance of childbearing, and child as a factor for stabilizing the marital life.

#### 3.2.1 Compulsory acceptance of childbearing

Some participants stated that they had to accept the pregnancy and fight the desire to abort the child, despite their unwillingness to do so. The reasons for this compulsory acceptance includes their religious beliefs that forbids abortion, and a fear of the consequences that the abortion may have on their fertility in the future, as well as their family's insistence on having the child.

*"When I found I'm pregnant*, *I did not believe it at all but I did not have any choice* … *At first*, *I did not want to be pregnant*, *but after pregnancy*, *I could not abort the child because (according to Islamic law) it is a sin*.*"*(Participant 17)

*"My father loves boys and asked me not to abort*. *Though I was determined to abort him*, *I had to keep him because of my parents and especially my husband's insistence*.*"* (Participant 21)

#### 3.2.2 Child as a factor for stabilizing the marital life

The adolescents in this study believe that having a child plays an important role in stabilizing and strengthening their marital life. Therefore, at times, despite their own personal unwillingness to have a child, they agree to do so in order to have a healthier marriage:

*"I have been married for two years*, *and a child is necessary for marital life*.*"*(Participant 15)

*"I want a child for my life*, *and every marital life needs a child*.*"*(Participant 18)

### 3.3 Hope and satisfaction

Most of the females expressed that accepting pregnancy brings about feelings of satisfaction and hope especially the females who accepted pregnancy actively; meanwhile others who accepted the pregnancy passively stated that they felt frustration and regret.

The adolescent females who felt satisfaction and hope expressed happiness at the prospect of motherhood and felt as if they were creating a purposeful and motivated life for themselves. This category included the following sub-categories:

#### 3.3.1 Hope to provide a better future for children

Some adolescents had lost life opportunities such as education and employment, due to an early marriage and pregnancy. Therefore, they hoped to compensate for this loss by providing a better future for their children.

*"I neglect myself and I just attempt to provide everything for my child*. *I would like to provide a better future for my child*.*"* (Participant 1)

*"I try to provide the things for my child that I did not have in my childhood*. *I would like my child to be more educated with a better job; I always think about these things*.*"* (Participant 22)

#### 3.3.2 Creating hope and motivation for life

Some adolescents mentioned that they have hope and motivation for their life as a child can eliminate their loneliness in the future:

*"When I wake up in the morning*, *from his kicking*, *I become more hopeful for the future of my life* …*; my life will become sweet by his birth*. *I am now busy with him*, *and the home is full of the baby’s sweet sound; all of them make life enjoyable*.*"* (Participant 5)

#### 3.3.3 Waiting for child birth and child care

Waiting for childbirth and caring for him/her was another theme, that emerged from the interviews. The participants considered this enjoyable, and stated that they felt motivated in their day to day life and anticipated the joys of childcare. After pregnancy, most of them have planned for child care:

*"I have a good feeling and I am in a hurry for the child’s birth*. *I am counting the days"* (Participant 2)

*"I'm glad and I'm enthusiastically waiting for his/her birth*, *I think of his/her face; when I see a child*, *I say when will my child be born*, *when will he/she walk or talk*.*"* (Participant 19)

#### 3.3.4 Happiness and satisfaction with the experience of motherhood

Most participants associate pregnancy with the feeling of motherhood. They stated that they even began experiencing these feelings by observing the passion and satisfaction of other mothers. Therefore, this led to the belief that their willingness and desire to experience motherhood for themselves motivated them to tolerate the difficulties of pregnancy.

*"I was happy because I wanted to be a mother and give birth to a baby*. *When I heard other mothers talking about their babies*, *I became interested in experiencing the same feeling*.*"* (Participant 18)

*"Well*, *it was a good feeling when I was informed that I am pregnant*. *This good feeling helps you tolerate the problems and difficulties of pregnancy*.*"* (Participant 9)

### 3.4 Frustration and regret

This category discusses the feelings of uncertainty and frustration, as well as the shock and regret some females feel with regard to their pregnancy. In other words, while feeling a general sense of happiness and satisfaction, these females also begin feeling frustrated due to the financial problems they begin to face as well as their lack of readiness to accept the role of motherhood, being too young for their first child, or being unable to take care of two children if they already have one.

#### 3.4.1 Feeling of uncertainty and desperation

Some participants experienced a sense of uncertainty and desperation as they felt that they are unprepared to take on and accept their maternal responsibilities due to a lack of information about child care. These feelings created a sense of doubt in these females and some even began considering abortion as an option:

*"Because my first child was so young*, *I wanted to abort the second one*, *but my family did not allow me*.*”* (Participant 12)

*"At first*, *I did not know; after two months*, *I felt I am pregnant*. *At first*, *I was happy*, *but later I was upset why did I listen to my husband and accept to get pregnant at this age*. *As I regretted it*, *I said "now how can I raise my baby*?*" It would have been better to get pregnant at least two years after marriage*.*"* (Participant 13)

#### 3.4.2 A sense of shock and regret

Some participants claimed that they were shocked and felt regret due to the unexpected nature of the pregnancy. They mentioned that this regret was as a result of the fear they felt at the prospect of being pregnant and the difficulties that came with it. Other females who were pregnant due to an egocentric decision, felt regret as they saw their husbands' reluctance with regard to having a child:

*"I could not believe it; I did not think of it at all*, *I was pregnant*! *(Surprised)* …*; pregnancy is good*, *but it has its own difficulties*, *too* …, *I tolerated a lot of problems and I felt regret*. *I did not want a child*.*"* (Participant 5)

*"It was unexpected and we were shocked* … *my husband did not believe it at all* …, *at first*, *my husband was confused and said it was a bit early but I said whatever God wants*.*"* (Participant 17)

#### 3.4.3 Economic barriers

Economic barriers and limited financial resources caused a sense of frustration and regret about the pregnancy for some of the participants. This type of financial regret was mostly seen among the mothers who were less religious compared to the other participants, or those who at the time, had other children:

*"I did not want to get pregnant at all because of financial problems…; I would like to give birth to several children if our financial situation was good because I love kids*.*"* (Participant 14)

*"In general*, *I love little kids; however*, *because of my condition*, *I wanted to abort the child*, *but I could not do that*.*"* (Participant 12)

## Discussion

The aim of the present study was to explore the perceptions of Iranian married female adolescents in confronting pregnancy. "Paradox of acceptance" emerged as the main theme of female adolescents in confronting pregnancy. During their pregnancy, teens experience ambivalent feelings that oscillate between a sense of willingness and unwillingness to remain pregnancy. One reason for these conflicting feelings is as a result of them defying the common social expectations of female adolescents, such as going to school and finding an income-generating job. Sadler, Novick and Oliver (2016) in a qualitative study on 30 Latina and African-American female pregnant adolescents (aged 14–25 years with different marital statuses) indicated that adolescents experience a range of paradoxical feelings (happiness to despair) when faced with pregnancy. For example, even though some adolescent mothers accepted their pregnancy after learning about their condition, their initial reaction was one of shock and confusion. A few female adolescents were discontent, experienced doubt, and feelings of desperation in confronting the maternal challenges [16]. Macutkiewicz & MacBeth (2017), in their review study of adolescent pregnancy intentions, identified that both positive and negative attitudes existed in adolescents. However, the positive feelings were predominant. Also, they suggested that adolescents display contradictory and incoherent attitudes towards their pregnancy that is in line with their developmental stage which is characterized by variability in their identity and beliefs [17]. In other words, the adolescents in the study exhibited dual perceptions when it came to accepting their pregnancy as a result of the stage of life they are currently in and the changeable attitude they have towards various events. The participants in the current study exhibit similar responses in that, even the adolescents that had accepted their pregnancy willingly still felt feelings of frustration and regret, and on the other hand, those who had accepted the pregnancy as a result of the influence of others also felt satisfaction and hope along with their negative feelings. In addition, one category of a phenomenological study (2016) from Iran on 11 married female pregnant adolescents (aged 14–19 years) was similar to the main theme of the present study. That phenomenological study showed that female adolescents had dual self-perceptions regarding pregnancy. Although the participants had positive self-perceptions’ on becoming pregnant most of the time, they also experienced negative self-perceptions about their own pregnancy [4].

Actively accepting pregnancy is one of the common experiences of young females in confronting pregnancy at adolescence. The active and egocentric acceptance of pregnancy is related to the feelings of responsibility they have for their children which in and of itself is as a result of these females' desire to experience the feelings of motherhood and child rearing. In a phenomenological study (2016) from Iran on married female pregnant adolescents demonstrated that the participants’ egocentric perceptions were intensified after becoming pregnant [4] which is in line with the findings in this study.

In the present study, some adolescents mentioned that they were forced to accept their pregnancy despite their unwillingness to have a child. They felt the role of motherhood was imposed on them as a result of their inability to decide freely, current social, religious, and family situations, as well as the guilt they faced if they aborted the child and the feeling that they had to submit to the will of God. Some studies reported similar results [12,18,19]. Unintended pregnancy is a common finding among adolescents [20–22]. According to the World Health Organization, every year more than 82 million unplanned adolescent pregnancies occur in low- and middle income countries [23]. Mohammadi, Montazeri, Alaghband-Rad, Ardabili & Gharacheh, (2016), in a phenomenological study from Iran, declared that all married female pregnant adolescents participating in the study considered pregnancy as a way to enter adulthood and grow up. Most of the young females believed that they were not ready to accept the roles and responsibilities of early pregnancy and motherhood physically, emotionally, and mentally, and that they were forced to accept pregnancy due to their family members' insistence [4]. Furthermore, some young females viewed pregnancy as a contributing factor to a stable and strong marital life. Loke & Lam (2014) found that stability in marital relations is the most effective factor in acceptance of pregnancy [19], which was confirmed by other studies too [24, 25]. However, this finding is inconsistent with the findings of Williams & Vines (1999); in the phenomenological study on pregnant female adolescents, they reported five themes including poor performance in the past, the disintegration of relationships, emotional separation, problem fixing, and reconnection. According to their findings, pregnancy was as a repackage for a new life for adolescents because early pregnancy has led to the disintegration of the marital life, and created an opportunity for reconnection [26]. In the current study, while not a dominant theme among the adolescents, one of the adolescents who had gotten pregnant despite the reluctance of her husband stated that her pregnancy created a distance between herself and her partner therefore negatively impacting her marriage. The reason for this difference is that in Iran, pregnancies occur within the marital relationship in accordance with the social and Islamic religion norms within the country. On the other hand, in the Williams and Vines study, most females were pregnant without being married to their partners. As these adolescents experience pregnancy within the context of the marriage, their perception of their success or failure as parents depends on the status of their relationship with their partner [27]. Another advantage of becoming pregnant while married is that married mothers receive social rewards for their pregnancy while unmarried mothers face many problems such as loneliness, poverty, and social isolation.

The paradox of acceptance is a theme that frames the experience of dealing with pregnancy in female adolescents and provides a better understanding of the inner struggle as well as the threats and opportunities that these adolescents experience. Generally, these adolescents viewed pregnancy as an opportunity for growth, stabilizing their marital life, and compensating for childhood and adolescent deficiencies, leading to a satisfying life in the future. At the same time, due to untimely pregnancy and an early undertaking of the maternal responsibilities, they had lost many occupational and educational opportunities, which play a major role in ensuring a good future for them. Some studies indicate that, while some mothers view their pregnancy as the beginning of a new and exciting chapter in their life, the pregnancy still presents several challenges for adolescents which leads these mothers to feel both happiness and frustration [19, 28]. For some adolescents, early pregnancy is viewed as a chance to mature and grow which contributes to their feelings of satisfaction and hope for the future. On the other hand, some adolescents feel frustrated and regretful due to their lack of access to material resources, family support, and sexual partners. [19]. Smith Battle & Leonard (1998) examined US adolescents experiences 4 years after the birth of their first child using a phenomenological approach and concluded that for some adolescents, early pregnancy means situating in a positive direction that reassures them about stability and a future full of hope; however, others feel reluctant and ambivalent about the pregnancy and parental acceptance [29]. Montgomery (2004) conducted a study by using the phenomenological approach to investigate the experiences of young females who had planned their pregnancy and found that planned pregnancy creates motivation for better and efficient performance [24]. The young females believed that their lives are difficult with pregnancy but they had decided to provide a better life for themselves and their child. Some adolescents emphasized the plans for the future of their child like buying clothes and opening a bank account.

Based on the current study, although some adolescents considered their pregnancy and their new found identity as a mother a positive event, due to a lack of preparation with regard to undertaking the responsibilities of child care, some adolescents found this event to be unexpected and difficult as well. The simultaneous occurrence and the challenges of events such as the transition to adulthood, marrying young, and an early pregnancy contributed to making the acceptance of pregnancy more difficult for these teens.

The adolescents in the current study mentioned that they felt frustrated as a result of the economic barriers they faced even though they had a personal desire and interest to become pregnant and experience motherhood. The results are congruent with the qualitative study in Maryland done by Tanner et al. (2013), in which the preparation of young females to accept pregnancy and childbearing was highly associated with their economic status rather than their emotional maturity and intellectual readiness [30]. Additionally, in the present study, the adolescents with strong religious beliefs were faced with a lesser paradox in relation to the acceptance of pregnancy. They controlled their frustration and regret by their belief that a child is a blessing. In other words, in Islam and the Persian culture in general, having a child is viewed as have several positive outcomes in one's life, some of which include continuing the path of the parents and helping them in time of need. Furthermore, in Islam a child is viewed as a blessing and as such the parents will be provided with a way to care for the child from God. Additionally, a good child will have lasting positive results for the parents in the after-life. Therefore, even adolescents who may not feel prepared to accept their pregnancy, eventually do so as a result of their religious belief system. A study showed that personal opinions and experiences related to childbearing, sexual experiences, and the social environment could create the paradox [19].

Adolescents who had a history of pregnancy and parenting were further faced with this paradox as a result of the additional financial strain and parenting responsibilities that this new child would bring. Yoo, Guzzo & Hayford (2014), using data from a nationally representative survey of unmarried young adults in Arizona, concluded that personal characteristics and experiences might influence the experience of this paradox as it may change the individual's perception of the positive and negative consequences of childbearing [31].

Finally, the results of this study indicated that the contrast between active and egocentric acceptance and passive and allocentric acceptance as well as the struggle between the sense of satisfaction and hope and frustration and regret resulted in creating the paradox of accepting pregnancy. In general, the adolescent mothers in this study had positive feelings toward pregnancy, and having a child was valuable for them despite the fact that the pregnancy might have created some problems for them. Therefore, due to the existing conflicting emotions, the adolescents never experienced a full rejection or acceptance of their pregnancy and the paradox of acceptance was consistently observed among them.

### Limitation

The main limitation of this study was that the participants were selected from among adolescents visiting PHCs or the selected hospital, meaning that the voices of adolescents who did not have antenatal care visits or chose not to participate are not represented. Also, this qualitative study was conducted in Rasht (Guilan Province, Iran). Therefore, like other qualitative studies, generalization of the results is limited; these studies should be repeated in different locations with different cultural and social conditions.

## Conclusion

The experience of pregnancy in adolescence is a phenomenon that is strongly influenced by the cultural, social, political, and religious contexts of every community. Generally speaking, as most studies focus on the experience of adolescent pregnancy outside the marital relationship, this study offers a new perspective on this phenomenon with regard to female pregnant married adolescents. These results can contribute to developing future mental and physical health programs tailored to the needs of adolescents.

According to the results of the study, given the dual-perceptions these adolescents have about their pregnancy acceptance, it is important that they have strong cognitive, emotional, and instrumental social support. This support should come from their families, husbands, health system, and government. Therefore, it is important that policy makers and health care providers design health promotion programs and develop mental and physical health promotion strategies tailored to the needs of adolescents. It is important to note, that this study was done with 24 married, female pregnant adolescents in only one city within a province in Iran making it a non-representative sample. Therefore similar studies should be done in the context of other cultures and religions to truly gain insight into this phenomenon.
